# Transcriptomic analysis reveals that *NIBAN1* overexpression is associated with BRAF^V600E^ mutation and increases the aggressiveness of thyroid cancer

**DOI:** 10.1016/j.gendis.2023.101094

**Published:** 2023-09-14

**Authors:** Paula Diana, Thaise Nayane Ribeiro Carneiro, Janete Maria Cerutti, Reginaldo Massanobu Kuroshu, Gianna Maria Griz Carvalheira

**Affiliations:** a Division of Genetics, Department of Morphology and Genetics, Escola Paulista de Medicina, Universidade Federal de São Paulo, São Paulo, Rua Botucatu, 740, Edifício Leitão da Cunha, 1 Andar, São Paulo, SP 04023900, Brazil; b Genetic Bases of Thyroid Tumors Laboratory, Division of Genetics, Department of Morphology and Genetics, Escola Paulista de Medicina, Universidade Federal de São Paulo, Pedro de Toledo 669, 11 Andar, São Paulo, SP 04039-032, Brazil; c Institute of Science and Technology, Universidade Federal de São Paulo, Av Cesare Mansueto Giulio Lattes, 1201, Eugênio de Mello, São José dos Campos, SP 12247-014, Brazil

*NIBAN1* overexpression has been reported in a wide range of thyroid carcinoma subtypes, and in other cancers, while it is not expressed in thyroid benign lesions and normal thyroid.^1^, ^2^, ^3^ However, the mechanism associated with its expression and prognostic value remains unclear. In this study, six independent cohorts were enrolled, in which *NIBAN1* expression was evaluated. A total of 583 patients with thyroid tumors, 326 patients with skin cancers, 293 patients with colorectal carcinoma, and 189 patients with lung carcinoma were analyzed. Our study demonstrates, for the first time, that *NIBAN1* expression varied according to thyroid tumor subtypes, presence of BRAF^V600E^ mutation, worse clinical features, and aggressive phenotype. Remarkably, the data suggest that BRAF^V600E^ mutation might influence *NIBAN1* expression in an MYC-dependent manner during the thyroid carcinogenic process. Furthermore, *NIBAN1* expression was predicted to be associated with stress-induced transcription/translation. In summary, our findings suggested that *NIBAN1* expression could be used not only to help preoperative diagnosis of a thyroid nodule but also may have prognostic implications.

*NIBAN1* expression levels were assessed in three differentiated thyroid tumors cohorts: (i) 52 thyroid samples, analyzed by RT-qPCR (RT-qPCR_BR) from the Brazilian cohort; (ii) 27 thyroid samples, assessed by RNA-Seq (RNA-Seq_BR) from the Brazilian cohort; and (iii) 504 patients with PTC from TCGA cohort (TCGA-THCA). In the RT-qPCR_BR and RNA-Seq_BR cohorts, *NIBAN1* expression was highest in classical PTC (CVPTC), followed by follicular carcinoma (FTC) and follicular subtype of PTC (FVPTC) (Fig. 1A, B). In the RNA-Seq_BR cohort, *NIBAN1* expression differed between CVPTC and FVPTC (Fig. 1B). In the TCGA-THCA cohort, its expression rate was prominently different between primary tumors and paired normal thyroid (Fig. S1), being higher in the tall subtype (TVPTC), intermediate in CVPTC, and lower in FVPTC (Fig. S2).

**Figure 1:**
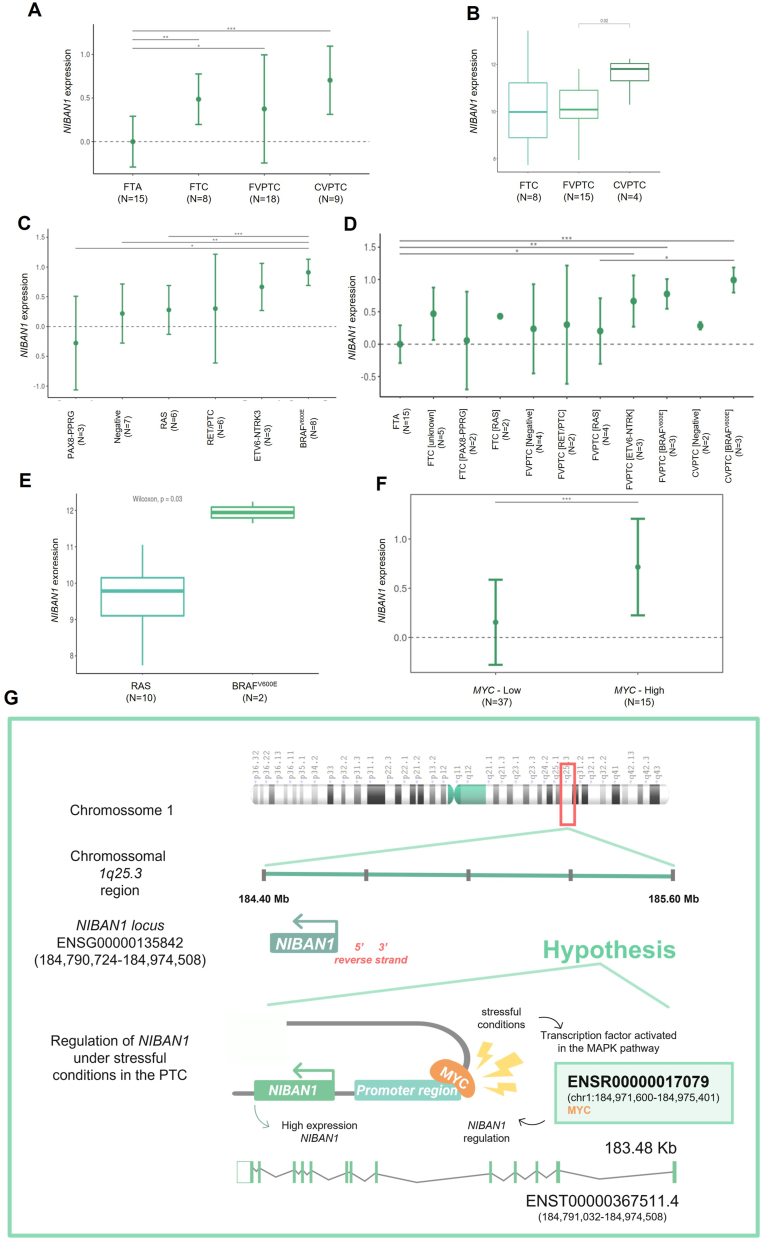
*NIBAN1* expression in relation to histological types, mutational profile, *MYC* expression, and the *NIBAN1* role in thyroid carcinomas. **(A)** RT-qPCR_BR cohort shows increased *NIBAN1* expression in thyroid carcinomas (FTC, FVPTC, and CVPTC) compared with FTAs (*P* < 0.05). **(B)** The expression of *NIBAN1* in the RNA-Seq_BR cohort shows differences according to the histological subtypes FVPTC and CVPTC (*P* = 0.02). **(C)** In the RT-qPCR_BR cohort, an increased expression of *NIBAN1* is detected in BRAF^V600E^ samples in relation to: K-N-HRAS; samples negative for these variants and PAX8-PPRG rearrangements (*P* < 0.05). **(D)** Samples were stratified by variants and histology subtypes, showing increased *NIBAN1* expression in CVPTC with BRAF^V600E^ compared with FVPTC, positive for K-N-HRAS variants, and FTAs. In FVPTC samples with BRAF^V600E^ variants and ETV6-NTKR fusion, *NIBAN1* expression is higher compared to FTAs (*P* < 0.05). **(E)** The RNA-Seq_BR cohort demonstrates the increase of *NIBAN1* expression in BRAF^V600E^ samples compared with K-N-HRAS (*P* = 0.03). **(F)** The RT-qPCR_BR cohort demonstrates the increase of *NIBAN1* expression in *MYC*-High samples compared with *MYC*-Low (*P* = 0.00015). **(G)** Schematic representation of the human chromosome 1 that harbors the *NIBAN1 locus* and its possible regulation by MYC. The *NIBAN1* gene (ENSG00000135842, 184,790,724–184,974,580), at 1q25.3, is transcribed by the reverse strand (antisense). In response to cellular stress, the MYC transcription factor may be activated, thereby increasing *NIBAN1* expression. The asterisks indicate significance values; ^∗^*P* < 0.05, ^∗∗^*P* < 0.01, ^∗∗∗^*P* < 0.001. The data is represented as mean ± standard deviation of RT-qPCR and RNA-Seq values (Log); *P* represents the statistical analysis from the nonparametric Wilcoxon and Kruskall–Wallis tests.

Moreover, the *NIBAN1* expression combined with the mutational profile revealed the presence of distinct subgroups. In the RT-qPCR_BR cohort, *NIBAN1* expression was higher in PTC with BRAF^V600E^ compared with those with *RAS* mutation, followed by those negative for known driver mutations and *PAX8-PPRG* fusions, respectively (Fig. 1C). The stratification of these samples according to histological subtypes and mutational profiles showed the highest expression of *NIBAN1* in CVPTC and FVPTC with BRAF^V600E^ or *ETV6-NTRK3* fusion compared with FTC and FVPTC with different mutational profile. A noticeable difference was observed between FVPTC BRAF^V600E^ and FVPTC *RAS* (Fig. 1D). Similar findings were observed in the RNA-Seq_BR cohort (Fig. 1E) and in the TCGA-THCA cohort (Fig. S3). When all PTC were grouped, *NIBAN1* expression was consistently higher in all PTC BRAF^V600E^ variants than those with *K-N-HRAS* (Fig. S4). To investigate the influence of *BRAF* and *RAS* mutations on *NIBAN1* expression, three TCGA cohorts, with high prevalence of *BRAF* mutations, were evaluated (Fig. S5). *NIBAN1* expression differed in colon (TCGA-COAD) and lung adenocarcinomas (TCGA-LUAD) cohorts (Fig. S6).

Since *NIBAN1* expression varies according to the mutational profile, its expression in TCGA-THCA, classified as either BRAF-Like or RAS-Like, was explored in all PTC samples with other mutations. This classification was built because the BRAF^V600E^ and RAS variants were mutually exclusive, allowing the discrimination of PTC in BRAF-Like or RAS-Like expressions, according to the 71-gene expression signature.^4^ Thus, it was observed that *NIBAN1* expression was higher in BRAF-Like samples compared with RAS-Like, regardless of the PTC histological subtype (Fig. S7).

To verify if the clinicopathological features could be related to *NIBAN1* expression, the TCGA-THCA samples were stratified according to the TNM stage. The results showed that *NIBAN1* expression is higher in more aggressive tumors (Fig. S8).

To create molecularly and biologically homogeneous subsets, the TCGA-THCA cohort was also stratified according to *NIBAN1* expression in two subsets: high (*NIBAN1*-High) and low (*NIBAN1*-Low) profiles (Fig. S9), and their molecular and clinical features were also evaluated (Fig. S10). The incidence of the *BRAF* mutation is higher in *NIBAN1*-High than in *NIBAN1*-Low, while RAS mutations are more prevalent in *NIBAN1*-Low than in *NIBAN1*-High (Fig. S11A). Remarkably, the HRAS mutation was detected exclusively in 13% of *NIBAN1*-Low (Fig. S11A). Both *NIBAN1* subsets contain PTC carrying other mutations (*Other*), corresponding to 56% in *NIBAN1*-Low and 31% in *NIBAN1*-High (Fig. S11B). Samples with *Other* mutations are composed of 11% of BRAF-Like profile and 89% RAS-Like, in *NIBAN1*-Low (Fig. S11C) and 45% of BRAF-Like profile and 55% RAS-Like, in *NIBAN1*-High (Fig. S11C). Knowing the importance of the mutational profile for the clinical phenotypes of thyroid tumors, the next step was to analyze the subsets according to histopathological subtypes and clinicopathological features. The results show that the *NIBAN1* subsets differed across all these clinical parameters (Fig. S12).

Considering that the tumor microenvironment can recruit different types of immune cells, the fractions of infiltrating immune cells between *NIBAN1*-Low and *NIBAN1*-high were predicted using 22 immune cell types. The *NIBAN1*-High subset showed higher levels of all immune cell infiltration types compared with *NIBAN1*-Low (Fig. S13). These results suggested that it is possible that *NIBAN1* overexpression may be clinically useful for inferring responsiveness to immunotherapy.

According to the results demonstrated herein, *NIBAN1* expression seems to be associated with histopathological characteristics and aggressiveness of thyroid tumors. Thus, the next step was to verify *in silico* the predicted biological functions of *NIBAN1* gene. To predict *NIBAN1* functions and signaling pathways associated with its expression, the differentially expressed genes (DEGs) in the *NIBAN1*-High subset compared with *NIBAN1*-Low were analyzed. This analysis found 14,602 down-regulated and 468 up-regulated DEGs (Fig. S14). Functional enrichment analysis showed that the overexpressed genes seem to be closely related to the regulation of transcription involved in the G1/S transition of the mitotic cell cycle, cytoplasmic translational initiation, and other functions (Fig. S15). These DEGs were enriched in the ER to Golgi transport vesicles and nuclear matrix. On the other hand, the down-regulated genes seem to be closely related to the cellular response to copper and other metal ions, and the regulation of G1/S transition of mitotic cell cycle (Fig. S16). These results, analyzed in *NIBAN1*-High samples with BRAF^V600E^ mutation and BRAF-Like samples, corroborate the *NIBAN1* involvement in the G1/S transition of the mitotic cell cycle, as well as transcription and translation during the cell cycle described before in functional experiments from our group.^3^

As demonstrated herein, the most prevalent genotype profile in the samples with high *NIBAN1* expression was the BRAF^V600E^ mutation. Thus, it was evaluated *in silico* whether the transcription factors, activated downstream on the BRAF pathway in MAPK signaling, may regulate the *NIBAN1* expression. The results showed that the transcription factors JUNB, JUND, and MYC are predicted to bind in the promoter region of *NIBAN1*, while FOS appears to act as a proximal enhancer. Although JUNB, JUND, and FOS are annotated as *NIBAN1* regulators, only *MYC* has increased expression in the *NIBAN1*-High samples (Table S1). Importantly, MYC is deregulated across many human cancers, and it is a main regulator of gene transcription. Our group has previously shown that *MYC* is overexpressed in more aggressive thyroid carcinoma subtypes.^5^ To investigate the influence of MYC on *NIBAN1* expression, samples from the RT-qPCR_BR cohort were classified based on *MYC* expression levels (Log values). Samples were classified into *MYC*-Low (<0.7) and *MYC*-High (>0.7). The results showed, for the first time, that samples with *MYC*-High present higher levels of *NIBAN1* compared with samples with *MYC*-Low (Fig. 1F). Therefore, it is possible that MYC could be activated in *NIBAN1*-High/BRAF^V600E^ samples, increasing *NIBAN1* levels in response to cellular stress (Fig. 1G).

Overall, these results, together with the functional experiments from our group,^3^ provide a global genetics picture of *NIBAN1* expression in thyroid carcinogenesis and demonstrate a strong association between *NIBAN1* overexpression and poor clinical features and more aggressive phenotype. Furthermore, *NIBAN1* expression also appears to be associated with the mutational profile of the tumor. These results suggested that BRAF^V600E^ mutation may influence *NIBAN1* expression, corroborating that its high expression level may lead to more aggressive conditions. All these data corroborate the insights, described by Diana and Carvalheira,^3^ which point out that *NIBAN1* could be a strong biomarker candidate in a preoperative FNA diagnosis in thyroid lesions. Despite the promising results, this retrospective analysis must be further validated in a greater number of clinical cases and multicentric studies.

## Ethics declaration

The study followed the Declaration of Helsinki and was analyzed (ID: CEP/UNIFESP: 0008/2016, CEP/UNIFESP: 0781/2016) by the Clinical Research Ethics Committee of the Universidade Federal de São Paulo. Signed informed consent was obtained from each patient.

## Author contributions

Paula Diana and Gianna Maria Griz Carvalheira wrote the draft of the paper. Paula Diana, Reginaldo Massanobu Kuroshu, Thaise Nayane Ribeiro Carneiro, and Gianna Maria Griz Carvalheira designed the experiments. Paula Diana, Thaise Nayane Ribeiro Carneiro, Reginaldo Massanobu Kuroshu, Gianna Maria Griz Carvalheira, and Janete Cerutti analyzed the data. Paula Diana, Reginaldo Massanobu Kuroshu, and Gianna Maria Griz Carvalheira prepared the figures and table. Paula Diana, Thaise Nayane Ribeiro Carneiro, Janete Maria Cerutti, Reginaldo Massanobu Kuroshu, and Gianna Maria Griz Carvalheira reviewed and edited the manuscript.

## Conflict of interests

The authors declare no conflict of interests.

## Funding

This work was supported by Coordenação de Aperfeiçoamento de Pessoal de Nível Superior (CAPES) (N°. 88882.430337/2019-01); Fundação de Amparo à Pesquisa do Estado de São Paulo (FAPESP) (N°. 2014/06570-6 and 2015/04164-3) and Conselho Nacional de Desenvolvimento Científico e Tecnológico (CNPq) (N°. 470441/2013-5).
