# Supplementary material for: Transcriptomic analysis reveals that NIBAN1 overexpression is associated with BRAFV600E mutation and increases the aggressiveness of thyroid cancer
Source: Genes Dis. 2023 Sep 14;11(4):101094. doi: 10.1016/j.gendis.2023.101094 (PMC10904183; doi:10.1016/j.gendis.2023.101094)
Supplement: Multimedia component 1 [file mmc1.docx]

**ONLINE SUPPLEMENTARY MATERIAL**

**Contents**

**Supplementary Table 1: Genes activated in the MAPK pathway and their differential expression in the *NIBAN1*-High subset**

| **Table S1:** Genes activated in the MAPK pathway and their differential expression in the *NIBAN1*-High subset | | | | | | | |
| --- | --- | --- | --- | --- | --- | --- | --- |
| **ID.ENSEMBL** | **Symbol** | **baseMean** | **log2 Fold Change** | **lfcSE** | **stat** | **p-value** | **padj** |
| ENSG00000136997 | MYC | 1174.6192 | 1.3307024 | 0.13662751 | 9.739637 | 2.042821e-22 | 3.164025e-21 |
| ENSG00000170345 | FOS | 44705.476 | -0.6030830 | 0.17289453 | -3.488155 | 4.863657e-04 | 1.071968e-03 |
| ENSG00000130522 | JUND | 16779.114 | -0.8179573 | 0.1152925 | -7.094627 | 1.297007e-12 | 7.557596e-12 |
| ENSG00000171223 | JUNB | 18498.4 | -0.165671 | 0.109552 | -1.51226 | 0.130469 | 0.183171 |
