# Supplementary material for: Transcriptomic analysis reveals that NIBAN1 overexpression is associated with BRAFV600E mutation and increases the aggressiveness of thyroid cancer
Source: Genes Dis. 2023 Sep 14;11(4):101094. doi: 10.1016/j.gendis.2023.101094 (PMC10904183; doi:10.1016/j.gendis.2023.101094)
Supplement: Multimedia component 2 [file mmc2.docx]

**ONLINE SUPPLEMENTARY MATERIAL**

**Contents**

**Supplementary Figure 1** In the TCGA-THCA cohort, *NIBAN1* expression has increased in primary PTC tumors compared to the respective normal tissues (*P* = 7.8e-07).

**Supplementary Figure 2** *NIBAN1* expression differs between PTC histopathological subtypes from the TCGA-THCA cohort (*P* = 5e-11).

**Supplementary Figure 3** BRAF^V600E^ and histopathological subtypes. TCGA-THCA cohort shows increased *NIBAN1* expression in histopathological subtypes with BRAF^V600E^ variants when compared to subtypes with K-N-HRAS (P = 6.1e-12). *P* represents the statistical analysis from the nonparametric Wilcoxon and Kruskall-Wallis tests, respectively.

**Supplementary Figure 4** In TCGA-THCA cohort, *NIBAN1* has increased expression in samples positive for BRAF^V600E^ variants compared to K-N-HRAS (*P* = 1.3e-13)

**Supplementary Figure 5** Frequency of *BRAF* and *K-N-HRAS* mutations in TCGA tumors.

**Supplementary Figure 6** *NIBAN1* expression in melanoma, colon and lung, respectively, in relation to mutational profile of BRAF and K-N-HRAS.

**Supplementary Figure 7** BRAF-Like and RAS-Like expression profile in relation to *NIBAN1* expression in PTC of TCGA-THCA.

**Supplementary Figure 8** *NIBAN1* expression in relation to clinicopathological features in PTC of TCGA-THCA.

**Supplementary Figure 9** Sample stratification by the quartile method.

**Supplementary Figure 10** Molecular and clinicopathological characteristics of the *NIBAN1*-Low and *NIBAN1*-High subsets.

**Supplementary Figure 11** Mutational profile in the *NIBAN1*-Low and *NIBAN1*-High subsets.

**Supplementary Figure 12** Clinicopathological characteristics of tumor samples stratified into *NIBAN1*-Low and *NIBAN1*-High.

**Supplementary Figure 13** Presence of infiltrating in the *NIBAN1*-Low and *NIBAN1*-High subsets.

**Supplementary Figure 14** Detection of DEGs in the *NIBAN1*-Low and *NIBAN1*-High.

**Supplementary Figure** **15** Prediction of the functions and cellular component of the overexpressed genes in the *NIBAN1*-High subset from TCGA-THCA cohort.

**Supplementary Figure S16** Prediction of the functions and cellular component of the underexpressed genes in the *NIBAN1*-High subset from TCGA-THCA cohort.


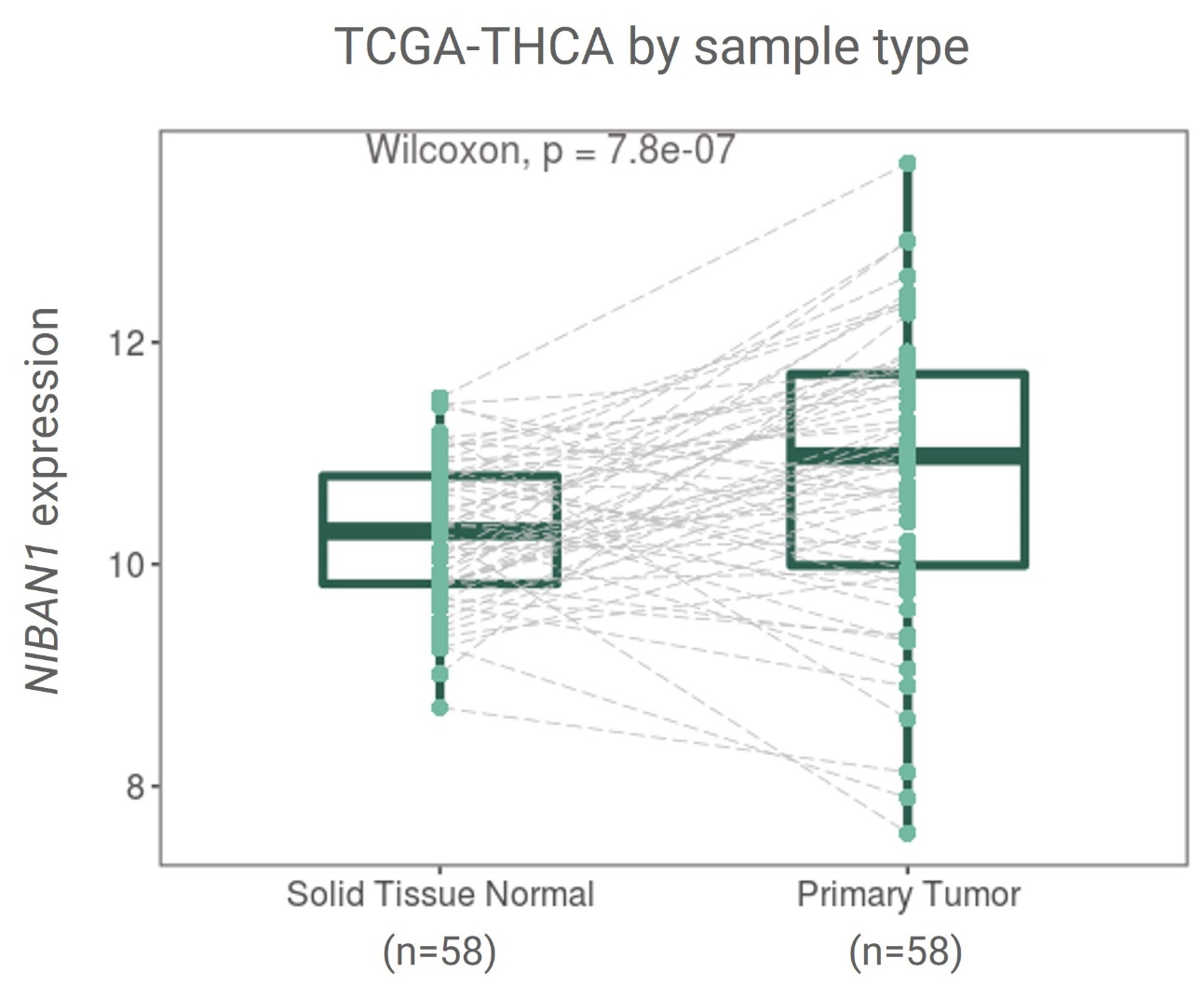


**Figure S1** In the TCGA-THCA cohort, *NIBAN1* expression has increased in primary PTC tumors compared to the respective normal tissues (*P* = 7.8e-07).


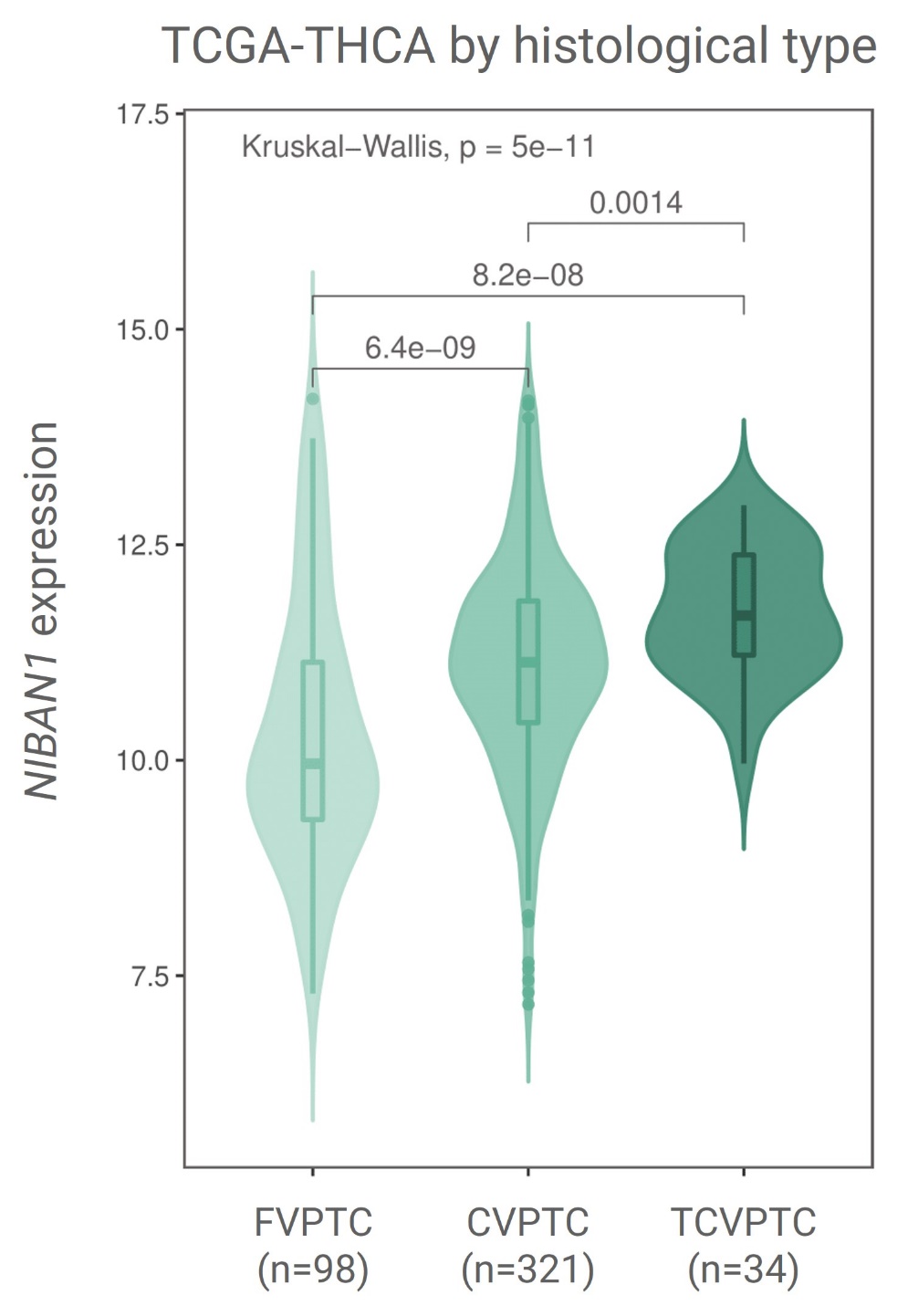


**Figure S2** *NIBAN1* expression differs between PTC histopathological subtypes from the TCGA-THCA cohort (*P* = 5e-11).


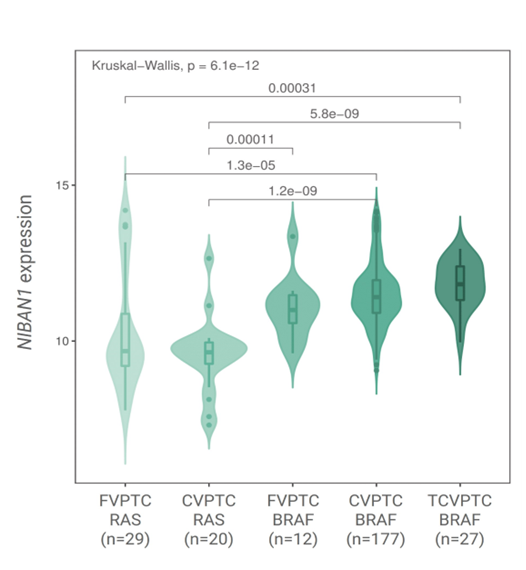


**Figure S3** BRAF^V600E^ and histopathological subtypes. TCGA-THCA cohort shows increased *NIBAN1* expression in histopathological subtypes with BRAF^V600E^ variants compared to subtypes with K-N-HRAS (P = 6.1e-12). *P* represents the statistical analysis from the nonparametric Wilcoxon and Kruskall-Wallis tests, respectively.

**
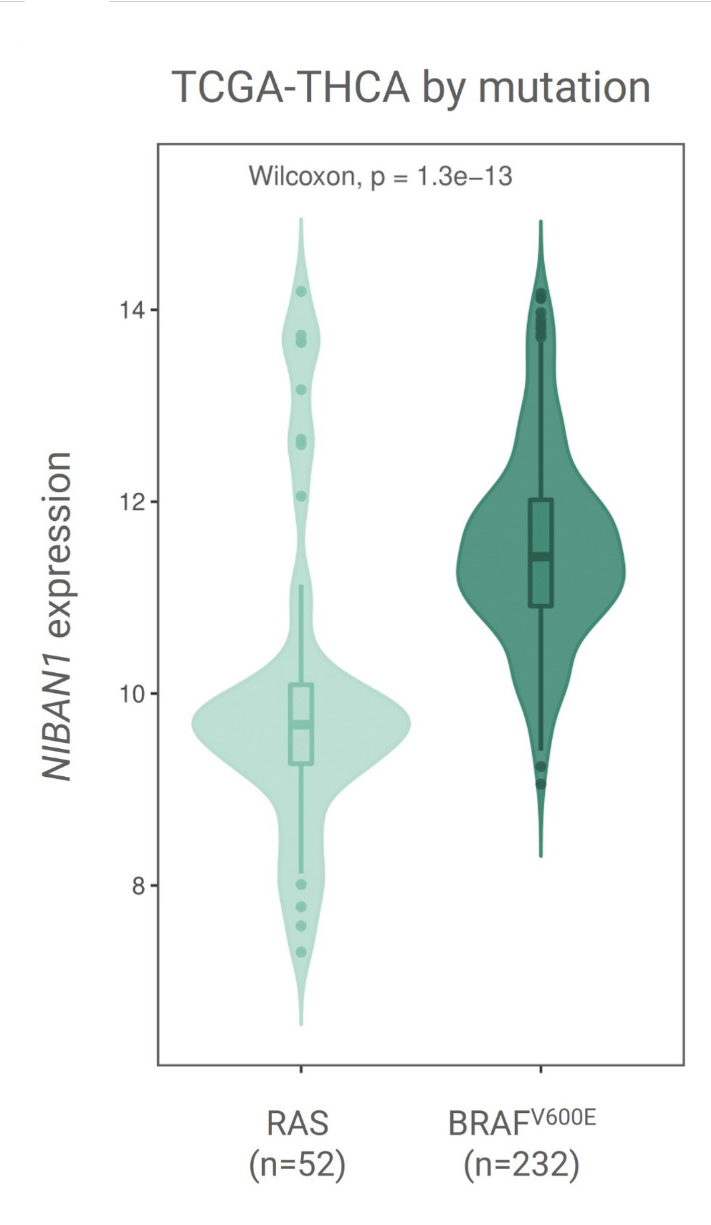
**

**Figure S4** In TCGA-THCA cohort, *NIBAN1* has increased expression in samples positive for BRAF^V600E^ variants compared to K-N-HRAS (*P* = 1.3e-13)

**
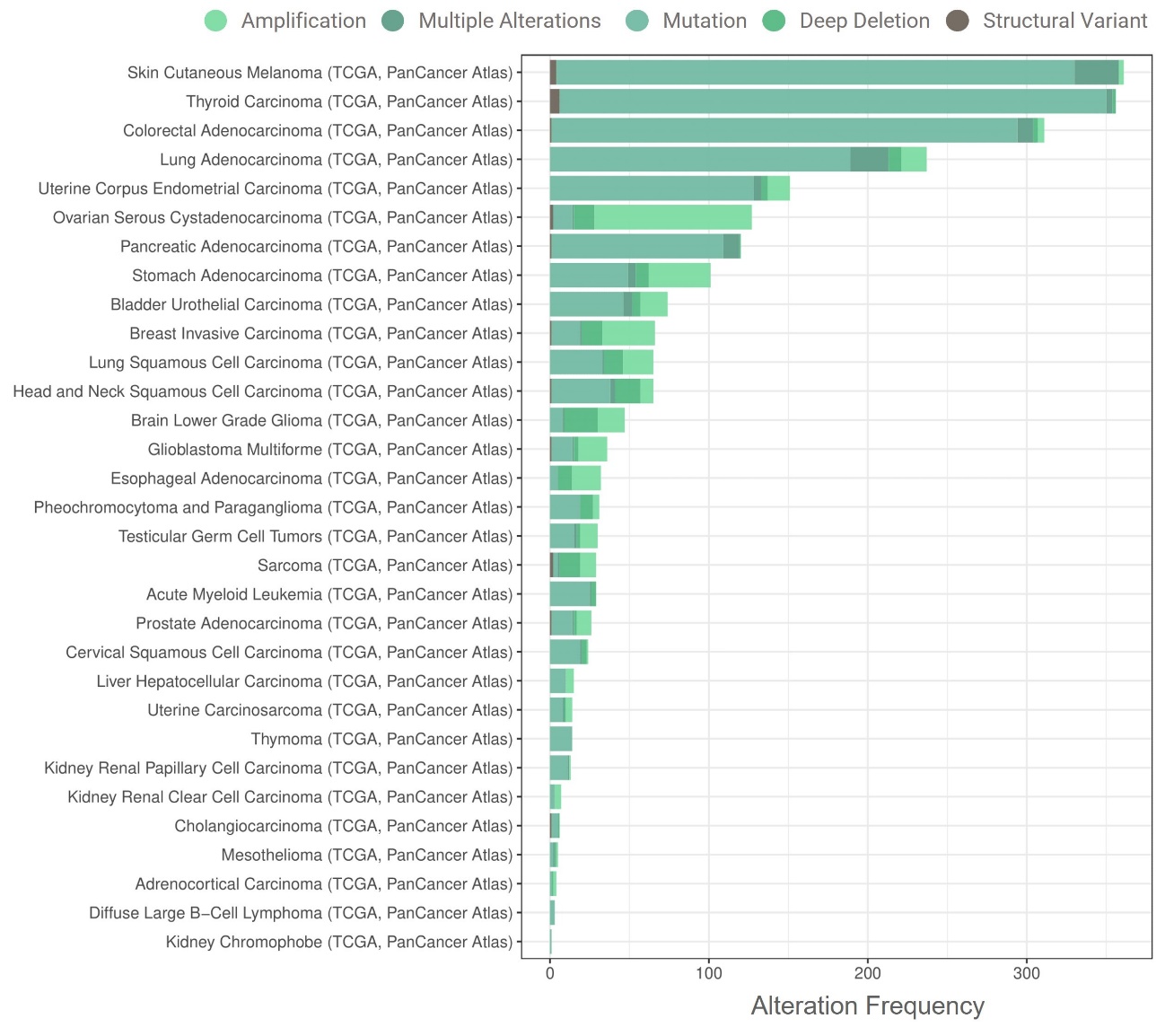
Figure S5** Frequency of *BRAF* and *K-N-HRAS* mutations in TCGA tumors.

**
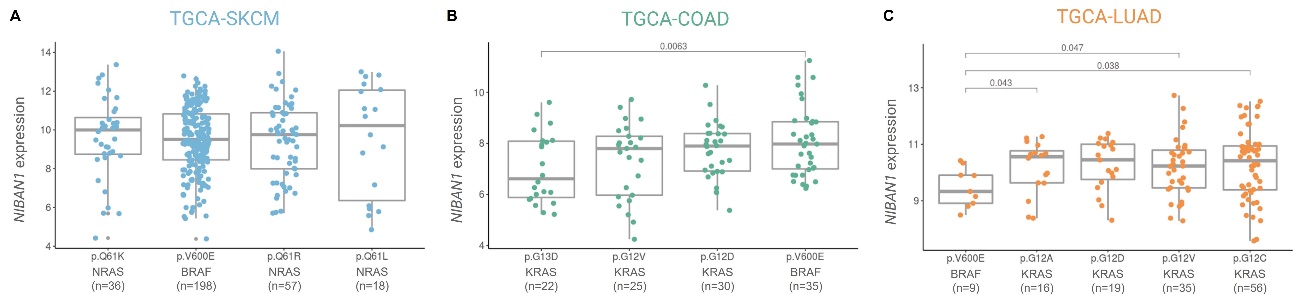
**

**Figure S6** *NIBAN1* expression in melanoma, colon and lung, respectively, in relation to mutational profile of *BRAF* and *K-N-HRAS*. In TCGA-SKCM, *NIBAN1* expression did not differ between samples with BRAF^V600E^ variants and NRAS (*P* >0.05). (A) In TCGA-COAD, *NIBAN1* expression is increased in samples with BRAF^V600E^ variants relative to samples with KRAS (p.G13D) (*P*= 0.0063). (B) In TCGA-LUAD, *NIBAN1* expression is increased in all samples with KRAS variants (p.G12A, *P*=0.043; p.G12D *P*=0.047; p.G12C *P*=0.038) compared to samples with BRAF^V600E^ (*P* <0.05). (C) Abbreviations: TCGA, The Cancer Genome Atlas; SKCM, Cutaneous Melanoma; COAD, Colon Adenocarcinoma; LUAD, Lung Adenocarcinoma. Data is represented as mean ± SD of RNA-Seq values (Log), *P* represents the statistical analysis from the nonparametric Wilcoxon test.

**
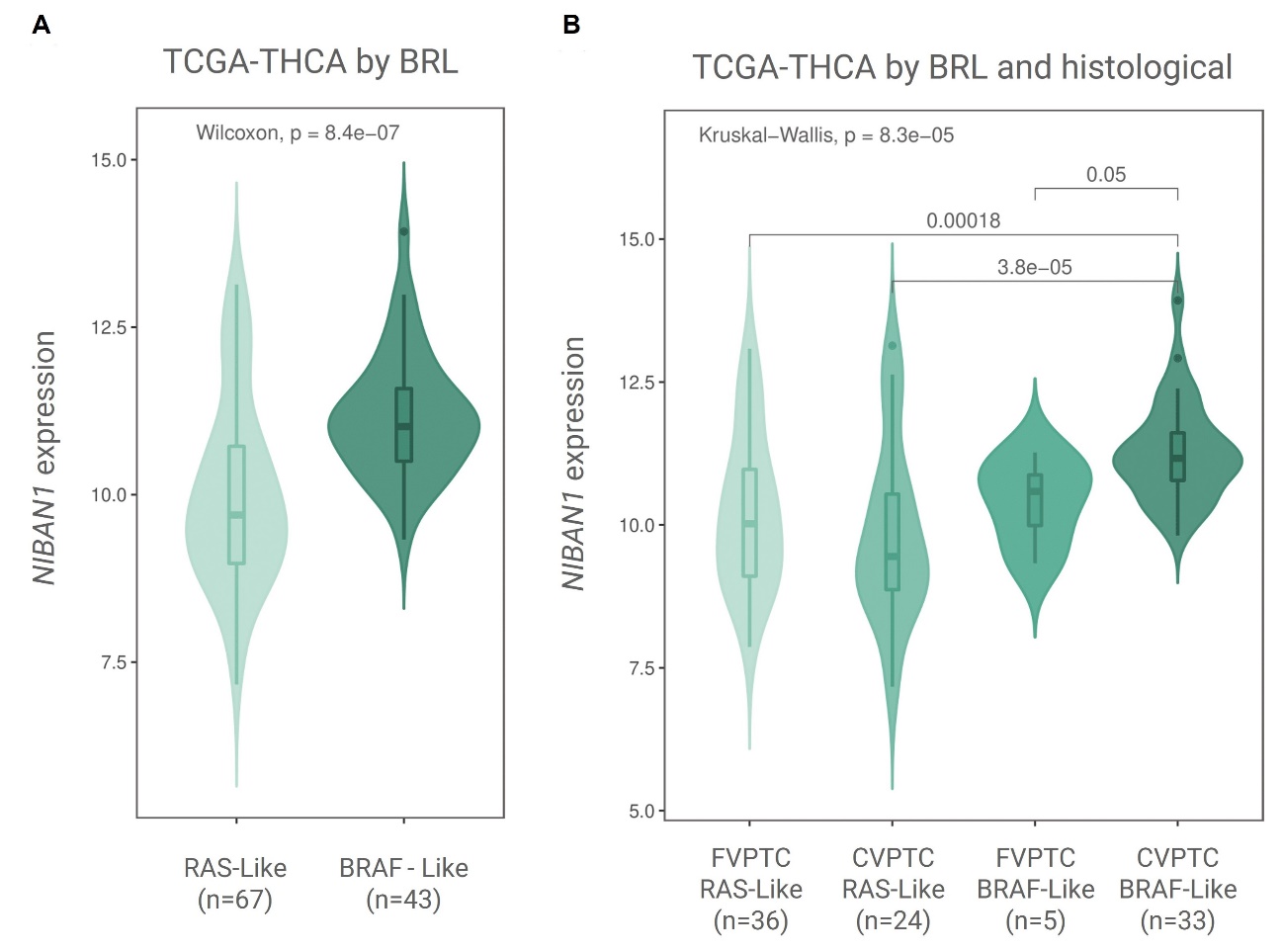
**

**Figure S7** BRAF-Like and RAS-Like expression profile in relation to NIBAN1 expression in PTC of TCGA-THCA. (A) NIBAN1 has increased expression in samples with BRAF-Like expression profile compared to RAS-Like samples (P = 8.4e-07). (B) NIBAN1 has increased expression in histopathological subtypes FVPTC and CVPTC with BRAF-Like expression profile compared to FVPTC and CVPTC subtypes with RAS-Like expression profile (P = 8.3e-05).  NIBAN1 expression data is presented as mean ± SD of RNA-Seq values (Log), P represents the statistical analysis from the tests Wilcoxon and Kruskall-Wallis parameters, respectively.


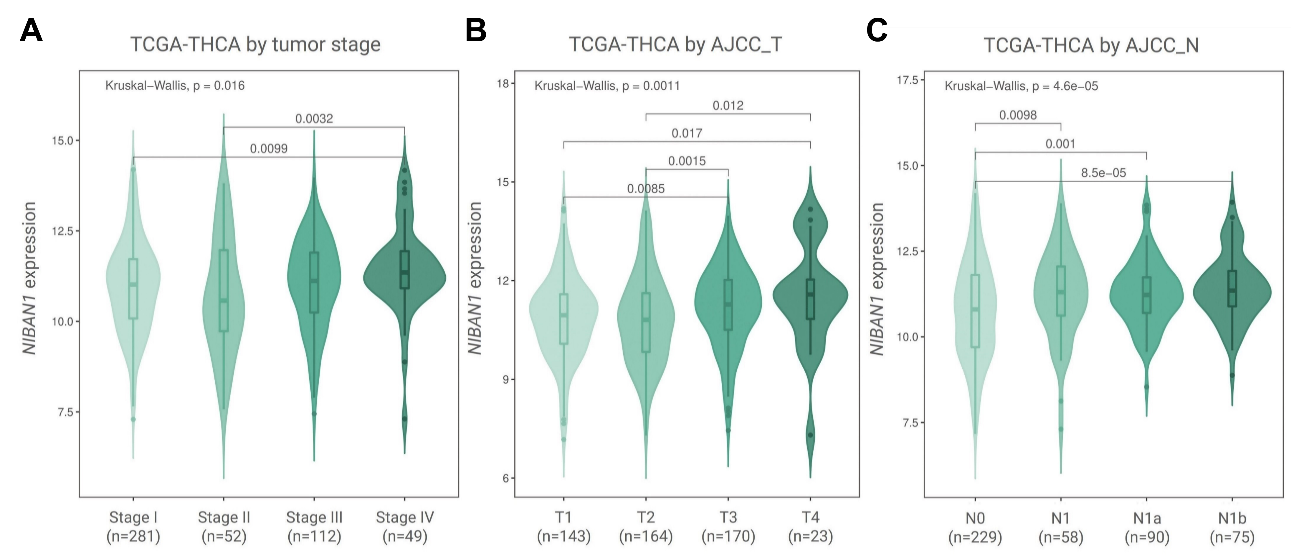


**Figure S8** *NIBAN1* expression in relation to clinicopathological features in PTC of TCGA-THCA. (**A**) *NIBAN1* has an increased expression in stage IV compared to stages I and II (*P* = 0.016). (**B**) *NIBAN1* has an increased expression in larger tumors (T3 and T4) (*P* = 0.0011). (**C**) *NIBAN1* has an increased expression when tumor spreads to regional lymph nodes (*P* = 4.6e-05). *NIBAN1* expression data is presented as mean ± SD of RNA-Seq values (Log), *P* represents the statistical analysis from the nonparametric Kruskal-Wallis test.

**
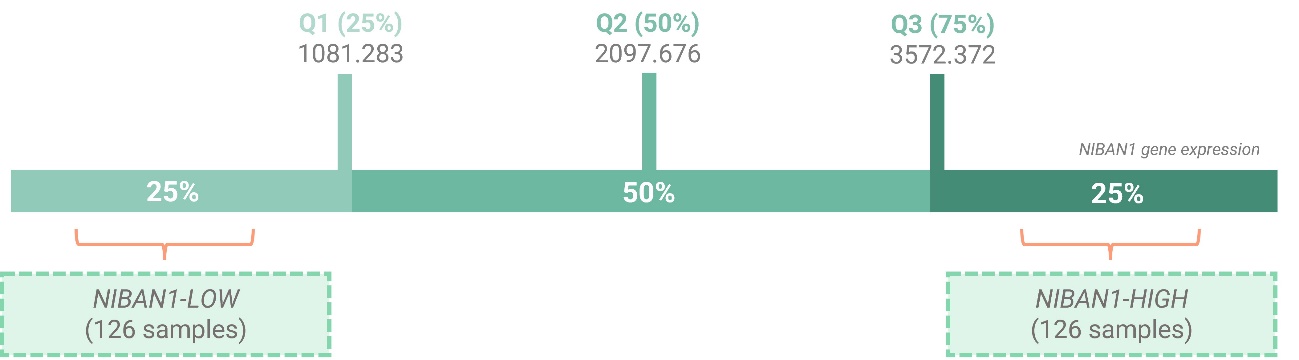
**

**Figure S9** Sample stratification by the quartile method. The first quartile (Q1) is characterized by the minimum expression of *NIBAN1* (25%), followed by the second quartile (Q2) that represents the median of the expression of *NIBAN1* (50%). The third quartile (Q3) corresponds to the maximum expression of *NIBAN1* (75%).

**
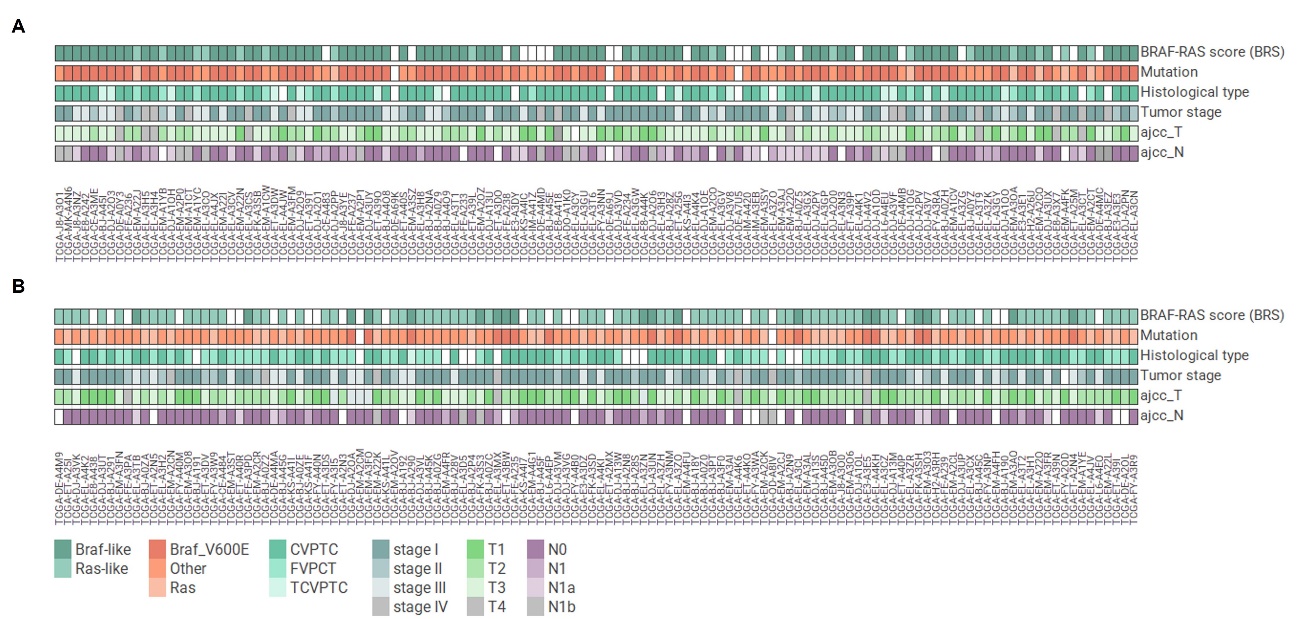
**

**Figure S10** Molecular and clinicopathological characteristics of the *NIBAN1*-Low and *NIBAN1*-High subsets. The samples that constitute *NIBAN1*-Low are represented in the upper portion (A) and *NIBAN1*-High in the lower portion (B). The quadrants demonstrate the distribution of the expression profile (BRAF-Like or RAS-Like), the mutational profile, the histological variants in PTC, as well as the staging, tumor size and presence of involvement lymph nodes. Abbreviation: AJCC, American Joint Committee on Cancer; T1, tumor ≤ 2 cm in greatest dimension limited to the thyroid; Stage I, the cancer is any size (Any T) and might or might not have spread to nearby lymph nodes (Any N); Stage II, the cancer can be any size (Any T). It might or might not have spread to nearby lymph nodes (Any N); Stage III, the cancer is any size and has grown extensively beyond the thyroid gland into nearby tissues of the neck, such as the larynx (voice box), trachea (windpipe), esophagus (tube connecting the throat to the stomach), or the nerve to the larynx (T4a); Stage IV, the cancer is any size and has grown extensively beyond the thyroid gland back toward the spine or into nearby large blood vessels (T4b); T2, tumor ≥2 cm but ≤ 4 cm in greatest dimension limited to the thyroid; T3, tumor ≥ 4 cm limited to the thyroid, or gross extrathyroidal extension invading only strap muscles; T4, Includes gross extrathyroidal extension into major neck structures; N0, no evidence of regional lymph nodes metastasis; N1, metastasis to regional nodes; N1a, metastasis to level VI or VII (pretracheal, paratracheal, or prelaryngeal/Delphian or upper mediastinal) lymph nodes, this can be unilateral or bilateral disease; N1b, metastasis to unilateral, bilateral, or contralateral lateral neck lymph nodes (Levels I, II, III, IV, or V) or retropharyngeal lymph nodes. The description of the TNM classification is based on the American Joint Committee on Cancer.

**
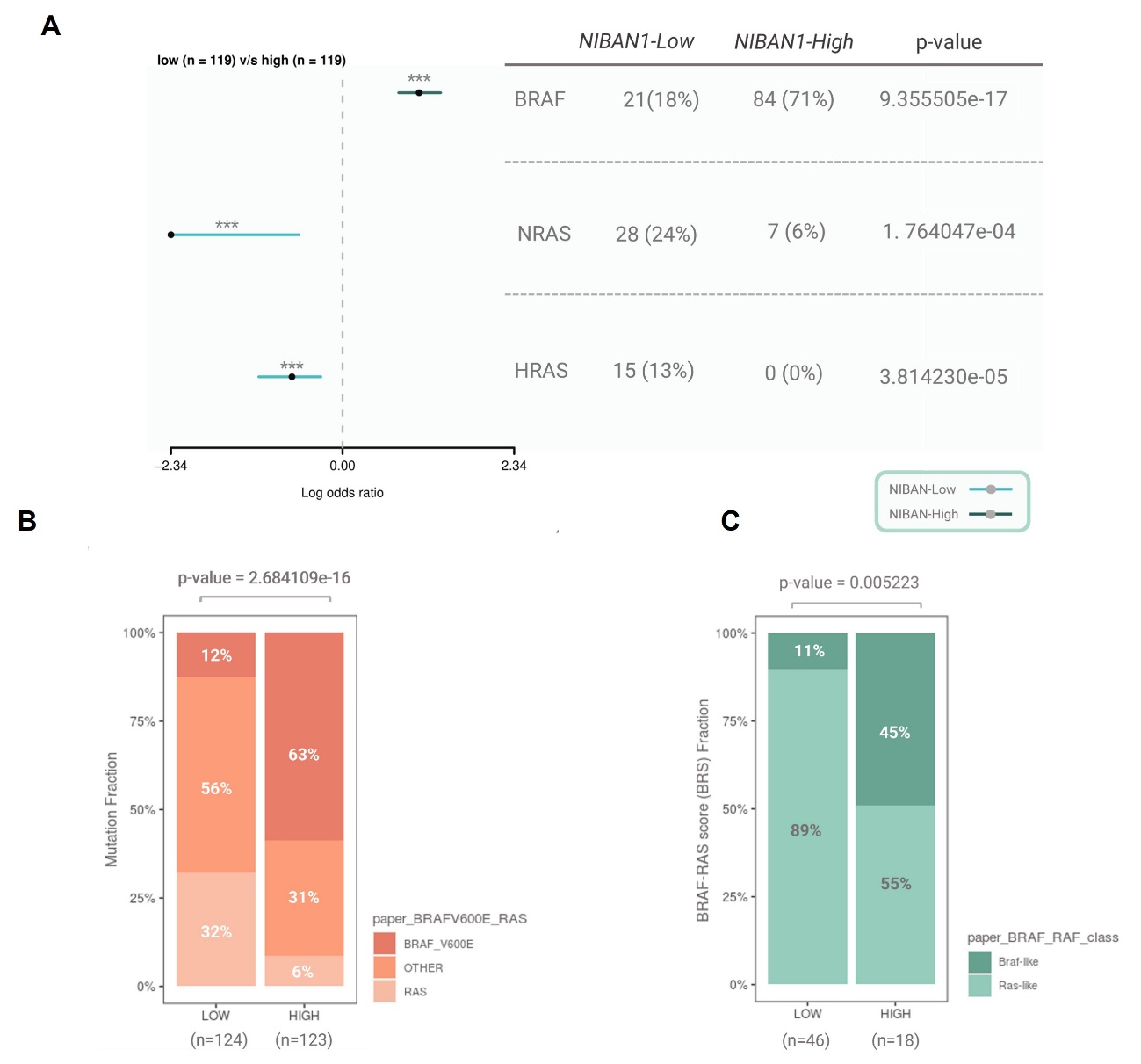
**

**Figure S11** Mutational profiles in the *NIBAN1*-Low and *NIBAN1*-High subsets. Blue lines represent mutations in *NIBAN1*-Low, in green recurrent mutations in *NIBAN1*-High. (**A**) Distribution of mutations in BRAF^V600E^, K-N-HRAS and less frequent variants, grouped in the *Other* group, in the two subsets *NIBAN1*-Low and *NIBAN1*-High (*P* = 2.544e-09). (**B**) Distribution of the BRAF-Ras-Like profile in the *Other* samples derived from the two subsets of *NIBAN1* (*P* = 0.003). (**C**) Asterisks indicate significance values * *P* < 0.05, ** *P* < 0.01, *** *P* < 0.001. Data is presented in percentage; *P* represents the statistical analysis of the *odds ratio* (**A**) and test statistic chi-square (*χ2*) (**B** and **C**)*.*


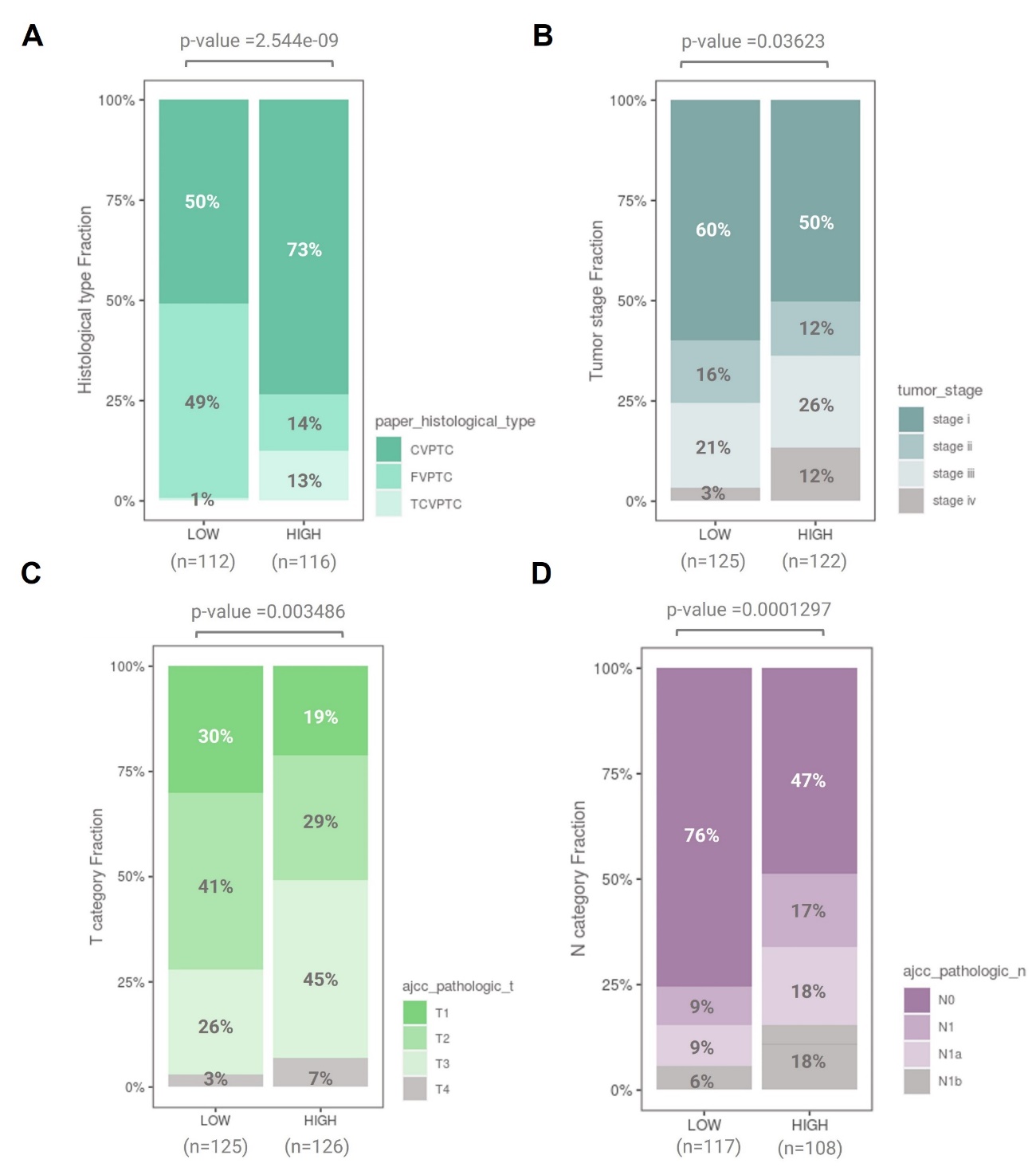


**Figure S12** Clinicopathological characteristics of tumor samples stratified into *NIBAN1*-Low and *NIBAN1*-High. (**A**) Distribution of histopathological subtypes in the two subsets of *NIBAN1* (P = 2.544e-09). (**B**) Distribution of tumor staging in the two *NIBAN1* subsets (P = 0.03623). (**C**) Tumor size (T) distribution in the two *NIBAN1* subsets (P =0.003). (D) Distribution of involvement for regional lymph nodes (N) in the two subsets of *NIBAN1* (P = 0.0001297). The description of the TNM classification is based on the American Joint Committee on Cancer. Data is presented in percentage and the P represents the analysis test statistic chi-square (χ2).


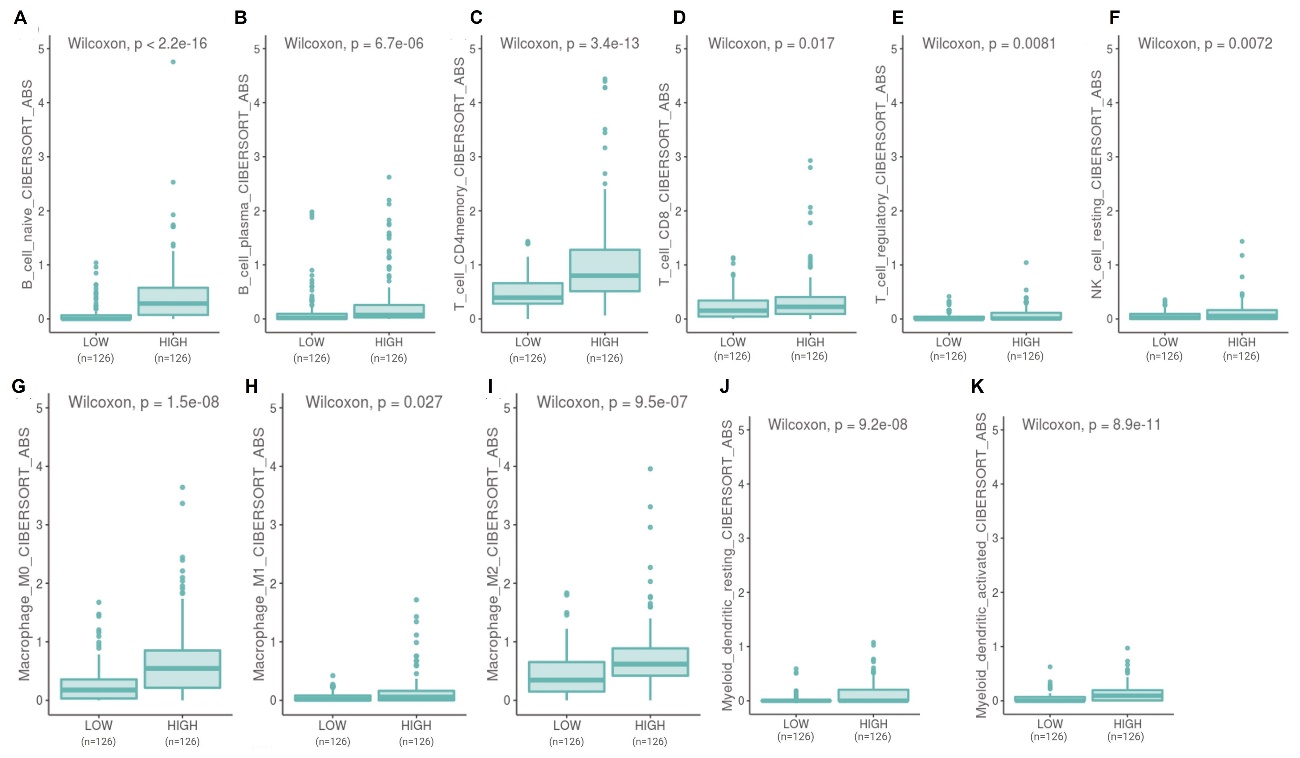


**Figure S13** Presence of infiltrating in the *NIBAN1*-Low and *NIBAN1*-High subsets. The *NIBAN1*-High subset showed differences compared to the *NIBAN1*-Low subset. These differences were evaluated in (**A**) immature B cells (*P* = <2.2e-16), (**B**) memory B cells (*P* = 6.7e-06), (**C**) memory T-CD4^+^ cells (*P* = 3.4e-13), (**D**) CD8^+^ T cells (*P* = 0.017), (**E**) regulatory T cells (*P* = 0.0081) (**F**) and NK cells (*P* = 0.0072), (**G**) M0 macrophages (*P* = 1.5e-08), (**H**), M1 macrophages (*P* = 0.027), (**I**) M2 macrophages (*P* = 9.5e-07), (**J**) resting myeloid dendritic cells (*P* = 9.2e-08), (**K**) and activated myeloid dendritic cells (*P* = 8.9e-11). *NIBAN1* expression data is presented as mean ± SD of RNA-Seq values (Log), *P* represents the statistical analysis from the nonparametric Wilcoxon test.


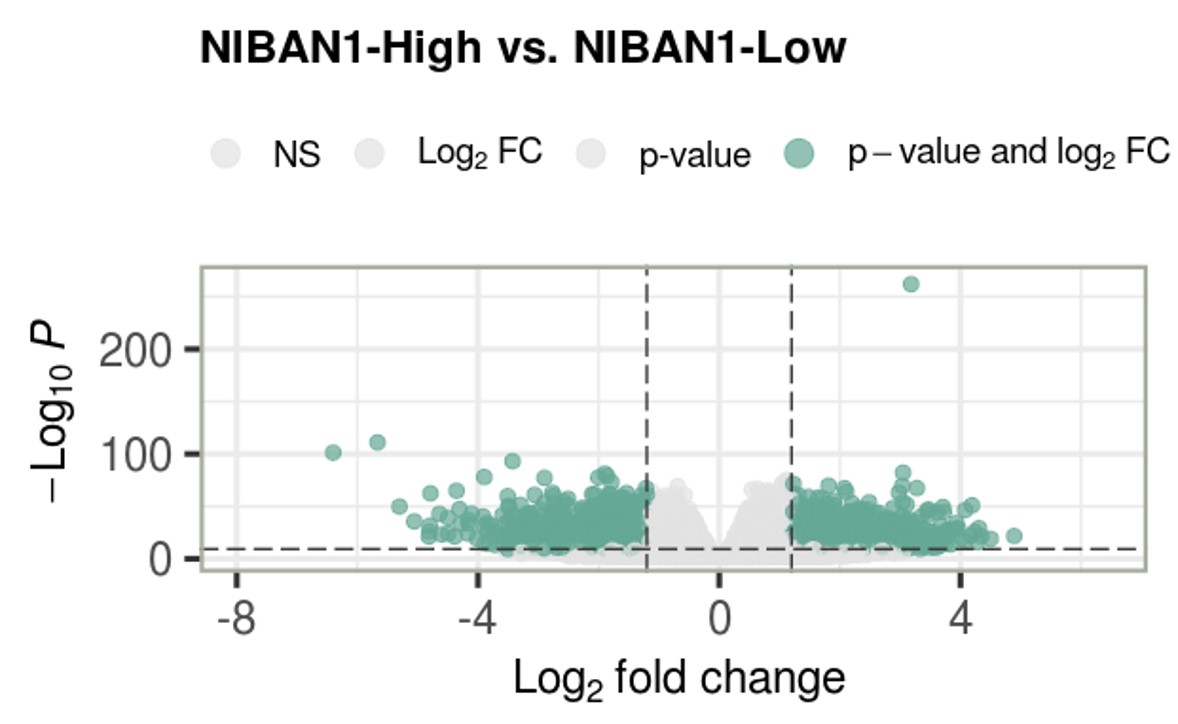


**Figure S14** Detection of DEGs in the *NIBAN1*-Low and *NIBAN1*-High. In green, the negatively regulated genes are represented on the left and the up-regulated genes on the right (*P* < 6e-10 | Log2 FC | > 1.3). Data is presented as a volcano graph. Log_10_ *P* and Log_2_ Fold Change represents the statistical analysis evaluated by the differential expression with DESEq. Abbreviation: DEG: Differentially Expressed Genes.

**
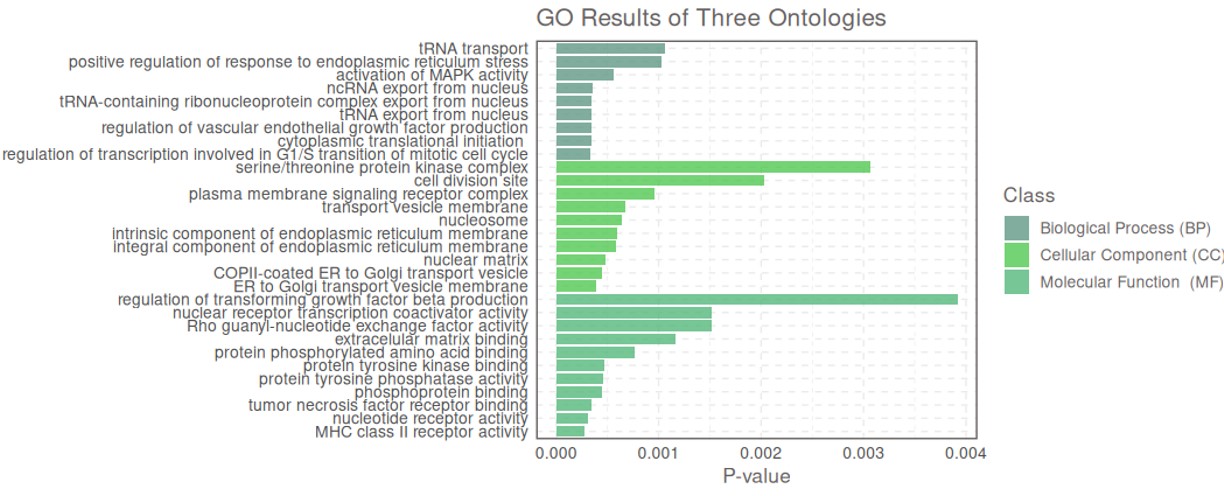
**

**Figure S15** Prediction of the functions and cellular component of the overexpressed genes in the *NIBAN1*-High subset from TCGA-THCA cohort.

**
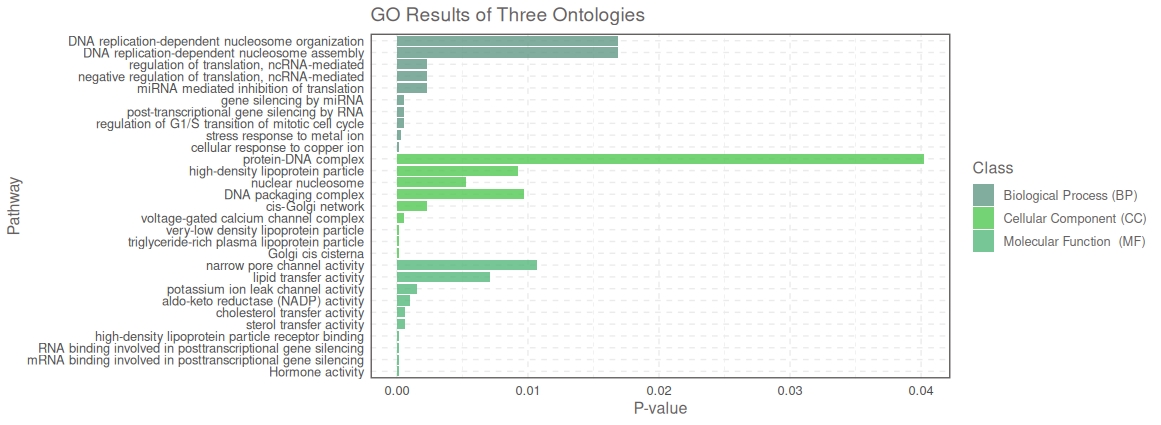
**

**Figure S16** Prediction of the functions and cellular component of the underexpressed genes in the *NIBAN1*-High subset from TCGA-THCA cohort.
