# Supplementary material for: Transcriptomic analysis reveals that NIBAN1 overexpression is associated with BRAFV600E mutation and increases the aggressiveness of thyroid cancer
Source: Genes Dis. 2023 Sep 14;11(4):101094. doi: 10.1016/j.gendis.2023.101094 (PMC10904183; doi:10.1016/j.gendis.2023.101094)
Supplement: Multimedia component 3 [file mmc3.docx]

**Materials and methods**

**Enrolled samples**

**Thyroid samples**

Thyroid tumors samples were composed of 79 Brazilian samples and 504 samples from TCGA. The Brazilian cohort was divided as followed: (i) 52 thyroid samples, analyzed by RT-qPCR (RT-qPCR_BR), ^1^ composed of nine classical subtypes (CVPTC) and 18 follicular subtypes (FVPTC) of PTC; 10 follicular carcinomas (FTC) and 15 follicular adenomas (FTA), and (ii) 27 thyroid samples, assessed by RNA-Seq (RNA-Seq_BR), ^2^ composed of four CVPTC; eight FTC, and 15 FVPTC. Altogether, the *NIBAN1* expression data (Log) was previously processed and normalized in other studies from our group. ^1,2^ This data has restricted access and is a part of the thyroid cancer data from the Laboratory “*As Bases Genéticas dos Tumores de Tireoide*”, from *Universidade Federal de São Paulo* (UNIFESP, *Campus* SP), coordinated by Professor Dr. Janete Maria Cerutti. The TCGA cohort enrolled 504 patients with PTC (TCGA-THCA). The analyzed RNA sequencing data comes from the level 2 of the TCGA-THCA that is accessible to the public and has an intermediate level of processing (htseq-count). This data consists of 60 normal solid tissue samples and 504 primary tumour samples. ^3^ This study was approved by Ethics Committee of *Universidade Federal de São Paulo*, UNIFESP.

**Extended analysis using additional TCGA samples**

Three cohorts obtained from TCGA cancer data and originated from different cancer subtypes were used for *NIBAN1* expression validation analysis. The TCGA-SKCM (Cutaneous Melanoma) with 326 samples; TCGA-COAD (Colorectal Adenocarcinoma) with 293 samples, and TCGA-LUAD (Lung Adenocarcinoma) with 189 samples were obtained from the UCSC Xena Portal (UCSC Xena, 2019, http://xena.ucsc.edu/). ^4^

**Data acquisition characteristics**

The data used was imported from TCGA to the R environment (version 4.0.2). RNA-Seq (GRCh38.p13) and the somatic mutation data was obtained with the TCGAbiolinks package (version 4.0). ^5^ The expression data was processed and normalized using the DESEq2 package. ^6^ Clinicopathological characteristics of thyroid tumors were analyzed according to American Joint Committee on Cancer/Tumor-Node-Metastasis Classification, ^3,7^ instead of the new 2022 WHO of thyroid tumors. ^8^

**Analysis of *NIBAN1* expression and mutational profile**

To assess the relationship between *NIBAN1* expression levels in tumor samples and the mutational profile of tumors, data from genotyping of the samples was used. Genotyping data was obtained from thyroid cancer samples from the Brazilian cohort and somatic variants from the TCGA project. ^1,2,3,9^ Expression levels from the RT-qPCR_BR cohort were previously calculated according to the method Comparative ΔΔCt. ^1^ FTAs were used as a control group. The TCGA mutation data was previously analyzed by the Maftools package R (version 4.1). ^10^

**Stratification of TCGA-THCA samples according *NIBAN1* expression: *NIBAN1*-Low and *NIBAN1*-High subsets**

The stratification was performed by measuring the quartiles of the expression of *NIBAN1*. The first quartile is the expression value that separates the lower 25% expression values of *NIBAN1* (*NIBAN1*-Low) from the upper 75% of the data (*NIBAN1*-High).

**Functional enrichment with gene ontology (GO)**

To infer the functions of the genes contained in the *NIBAN1*-High subgroup, differential gene expression (DEGs) analysis was performed with the DESeq2 package, ^6^ using the Benjamini-Hochberg method (*P* < 0.05). Functional enrichment was performed based on the Gene Ontology data, ^11,12^ which includes genetic functions at three levels: Biological Process (BP), Molecular Function (MF) and Cellular Component (CC). The ClusterProfiler package (version 4.0.2) ^13^ was used to detect the pathways and functions of DEGs.

**Prediction of cellular infiltrate in the *NIBAN1*-Low and *NIBAN1*-High subsets**

To infer the fraction of infiltrating immune cells in the *NIBAN1*-Low and *NIBAN1*-High subsets, the TIMER tool was used (version 2.0). ^14-16^ To infer these infiltrations, the method CIBERSORT ^17^ used a leukocyte gene signature matrix, called LM22.

**Prediction of transcription factors of *NIBAN1* gene**

To investigate the transcription factors of the *NIBAN1* gene, the GeneHancer-Regulatory Elements tool present in the GeneCards database (version 5.8) ^18^ was used.

**Analysis of *NIBAN1* expression in relation to *MYC* expression**

*MYC* expression levels were evaluated in the RT-qPCR_BR cohort. The *NIBAN1* expression data (Log) was previously processed and normalized, ^1^ while the *MYC* expression data (Log) was processed and normalized according to the method Comparative ΔΔCt. ^19^ According to *MYC* expression levels (Log values), the samples were classified into: (i) low when presented expression equal or less than 0.7, and (ii) high when presented expression greater than 0.7. Then, 37 samples were classified as low and 15 samples were classified as high.

**Statistical analyses**

The data is expressed as the mean ± standard deviation. All statistical analyses were performed via the statistical programming language R for Windows (cran.r-project.org). The relationship between *NIBAN1* expression and (i) histological types; (ii) mutational profile; (iii) clinicopathological features was analyzed by the non-parametric tests (Wilcoxon and Kruskall-Wallis tests). A two-tailed *P* less than 0.05 was considered statistically significant. For percentage of mutational profile in the *NIBAN1*-Low and *NIBAN1*-High subsets, *P* represents the statistical analysis of the *odds ratio* and test statistic chi-square (χ2). Clinicopathological characteristics of tumor samples stratified into *NIBAN1*-Low and *NIBAN1*-High were presented in percentage and the *P* represents the analysis test statistic chi-square (χ2). The relationship between the *NIBAN1*-Low and *NIBAN1*-High subsets and presence of infiltrating was analyzed by the non-parametric tests (Wilcoxon test). A two-tailed *P* less than 0.05 was considered statistically significant.

**References**

1. Carvalheira G., Nozima B. Heidi, Cerutti J. microRNA-106b-mediated down-regulation of C1orf24 expression induces apoptosis and suppresses invasion of thyroid cancer. *Database (Oncotarget)*. 2015.

2. Carneiro TNR, Bim LV, Buzatto VC, et al. Evidence of Cooperation between Hippo Pathway and RAS Mutation in Thyroid Carcinomas. *Cancers (Basel)*. 2021;13(10).

3. Network CGAR. Integrated genomic characterization of papillary thyroid carcinoma. *Cell*. 2014;159(3):676-90.

4. Goldman MJ, Craft B, Hastie M, et al. Visualizing and interpreting cancer genomics data via the Xena platform. *Nat Biotechnol*. 2020;38(6):675-678.

5. Colaprico A, Silva TC, Olsen C, et al. TCGAbiolinks: an R/Bioconductor package for integrative analysis of TCGA data. *Nucleic Acids Res*. 2016;44(8):e71.

6. Love MI, Huber W, Anders S. Moderated estimation of fold change and dispersion for RNA-seq data with DESeq2. *Genome Biol*. 2014;15(12):550.

7. Tuttle RM, Haugen B, Perrier ND. Updated American Joint Committee on Cancer/Tumor-Node-Metastasis Staging System for Differentiated and Anaplastic Thyroid Cancer (Eighth Edition): What Changed and Why? *Thyroid*. 2017;27(6):751-756.

8. Christofer Juhlin C, Mete O, Baloch ZW. The 2022 WHO classification of thyroid tumors: novel concepts in nomenclature and grading. Endocr Relat Cancer. 2023;30(2).

9. Bastos AU, Oler G, Nozima BH, Moysés RA, Cerutti JM. BRAF V600E and decreased NIS and TPO expression are associated with aggressiveness of a subgroup of papillary thyroid microcarcinoma. Eur J Endocrinol. 2015;173(4):525-40.

10. Mayakonda A, Lin DC, Assenov Y, Plass C, Koeffler HP. Maftools: efficient and comprehensive analysis of somatic variants in cancer. Genome Res. 2018;28(11):1747-1756.

11. Ashburner M, Ball CA, Blake JA, et al. Gene ontology: tool for the unification of biology. The Gene Ontology Consortium. Nat Genet. 2000;25(1):25-9.

12. Consortium GO. The Gene Ontology resource: enriching a GO ld mine. Nucleic Acids Res. 2021;49(D1):D325-D334.

13. Yu G, Wang LG, Han Y, He QY. clusterProfiler: an R package for comparing biological themes among gene clusters. OMICS. 2012;16(5):284-7.

14. Li B, Severson E, Pignon JC, et al. Comprehensive analyses of tumor immunity: implications for cancer immunotherapy. Genome Biol. 2016;17(1):174.

15. Li T, Fan J, Wang B, et al. TIMER: A Web Server for Comprehensive Analysis of Tumor-Infiltrating Immune Cells. Cancer Res. 2017;77(21):e108- e110.

16. Li T, Fu J, Zeng Z, et al. TIMER2.0 for analysis of tumor-infiltrating immune cells. Nucleic Acids Res. 2020;48(W1):W509-W514.

17. Chen B, Khodadoust MS, Liu CL, Newman AM, Alizadeh AA. Profiling Tumor Infiltrating Immune Cells with CIBERSORT. Methods Mol Biol. 2018;1711:243-259.

18. Fishilevich S, Nudel R, Rappaport N, et al. GeneHancer: genome-wide integration of enhancers and target genes in GeneCards. Database (Oxford). 2017.

19. Cerutti JM, Delcelo R, Amadei MJ, et al. A preoperative diagnostic test that distinguishes benign from malignant thyroid carcinoma based on gene expression. J Clin Invest. 2004;113(8):1234-42.
